# The Quality Improvement Demonstration Study: An example of evidence-based policy-making in practice

**DOI:** 10.1186/1478-4505-6-5

**Published:** 2008-03-25

**Authors:** Riti Shimkhada, John W Peabody, Stella A Quimbo, Orville Solon

**Affiliations:** 1 Institute for Global Health, University of California San Francisco, 50 Beale Street, Suite 1200, San Francisco, California, USA; 2 School of Economics, University of the Philippines, Diliman, Quezon City, Philippines

## Abstract

**Background:**

Randomized trials have long been the gold-standard for evaluating clinical practice. There is growing recognition that rigorous studies are similarly needed to assess the effects of policy. However, these studies are rarely conducted. We report on the Quality Improvement Demonstration Study (QIDS), an example of a large randomized policy experiment, introduced and conducted in a scientific manner to evaluate the impact of large-scale governmental policy interventions.

**Methods:**

In 1999 the Philippine government proposed sweeping reforms in the National Health Sector Reform Agenda. We recognized the unique opportunity to conduct a social experiment. Our ongoing goal has been to generate results that inform health policy. Early on we concentrated on developing a multi-institutional collaborative effort. The QIDS team then developed hypotheses that specifically evaluated the impact of two policy reforms on both the delivery of care and long-term health status in children. We formed an experimental design by randomizing matched blocks of three communities into one of the two policy interventions plus a control group. Based on the reform agenda, one arm of the experiment provided expanded insurance coverage for children; the other introduced performance-based payments to hospitals and physicians. Data were collected in household, hospital-based patient exit, and facility surveys, as well as clinical vignettes, which were used to assess physician practice. Delivery of services and health status were evaluated at baseline and after the interventions were put in place using difference-in-difference estimation.

**Results:**

We found and addressed numerous challenges conducting this study, namely: formalizing the experimental design using the existing health infrastructure; securing funding to do research coincident with the policy reforms; recognizing biases and designing the study to account for these; putting in place a broad data collection effort to account for unanticipated findings; introducing sustainable policy interventions based on the reform agenda; and providing results in real-time to policy makers through a combination of venues.

**Conclusion:**

QIDS demonstrates that a large, prospective, randomized controlled policy experiment can be successfully implemented at a national level as part of sectoral reform. While we believe policy experiments should be used to generate evidence-based health policy, to do this requires opportunity and trust, strong collaborative relationships, and timing. This study nurtures the growing attitude that translation of scientific findings from the bedside to the community can be done successfully and that we should raise the bar on project evaluation and the policy-making process.

## Background

While clinical interventions are typically grounded in scientific evidence before widespread adoption, public policies are not typically subjected to the same scientific rigor [1,2]. By definition public policies are made to affect whole populations [3]. This alone is a compelling reason to be judicious and well-informed before implementing new policy. The sheer costs of maligned policy add further to the gravity of choosing unwisely. Arguably, therefore, policy interventions should be put through the same level of caution and consideration as clinical trials, which do not nearly have the same scope or reach of social policies [4]. Increasingly, there is greater credence given to this argument and there is growing demand that decision-making in the policy environment be science-based [1,2,5-7].

There are a variety of scientific approaches for generating and finding the best evidence for diagnosing and treating disease, with the standard being the randomized clinical trial (RCT). In an RCT, the link between clinical care and health outcomes is often more direct than with policy change, thus causality is more readily evaluated and, for the most part, more easily measured. Generating randomized policy trials is more challenging in several important ways. Policy interventions can be difficult to randomize among populations, and challenges will arise when trying to time the introduction of interventions so that proper pre- and post- assessments are completed; similarly, measuring outcomes of interest is not trivial–collecting data of high caliber can not only be costly but often a significant lag time is required before population changes are seen. Additionally, interventions are rarely introduced devoid of political context, making it all the more difficult to separate science from the often contentious and fractious political environment. Perhaps one of the biggest limitations, however, is that policy interventions are often assumed to be effective when there is scant evidence that they are and, as a result, they are not even considered to be worthy of scientific investigation.

The Philippine Child Health and Policy Experiment, also known as the Quality Improvement Demonstration Study (QIDS), is a unique example of how a large randomized control longitudinal study can be introduced in a rigorous scientific manner to evaluate the impact of governmental policy interventions. In this paper, we describe how we were able to conduct a prospective randomized policy experiment, the lessons learned, and the few obstacles that remain.

## Methods

### Setting for the experiment

The Philippines Department of Health (DOH) took a major step towards improving the performance of the health sector in 1999 by launching the National Health Sector Reform Agenda (HSRA) to change the way health services were being financed and administered. The reform policies were directed mainly at a) increasing access to personal health services, especially for the poor, and b) improving the quality of care delivered at hospitals.

### Institutions

The Philippines Department of Health is the principal government agency that formulates national health policies and programs, and guides the development of local health systems, programs, and services. The DOH is led by the Secretary of Health, who is appointed by the President. An institution attached to the DOH, the Philippine Health Insurance Corporation (PhilHealth) is the governmental financial institution implementing the National Health Insurance Program, making it the largest insurance carrier in the Philippines. Mandated by the National Health Insurance Law of 1995, PhilHealth's priorities are to achieve universal coverage, expand insurance benefits, and ensure high quality of care and member satisfaction. The National Health Insurance Program covers employees from the formal sector, retirees and pensioners, self-employed individually paying individuals, and the poor. PhilHealth reimburses on a first-peso basis payment for inpatient services including room and board, surgical procedures, physician services, selected diagnostic and treatment procedures, and drugs. In 2006, PhilHealth had an estimated active membership coverage of 16 million members with more than 68 million beneficiaries, thus covering about 79% of the estimated 86.9 million in the entire country.

PhilHealth, however, has faced challenges in reaching their goals of universal coverage and improved health care delivery [8]. Recognizing that the ultimate success of reform policies lies in the understanding of how they affect the quality of health care provided to those in need and whether these changes have beneficial effects on health, 10 years ago PhilHealth committed itself to the financing and implementation of health reform policies targeted at improving health care for children and the poor.

The poor, also referred to as indigents in the Philippines, largely depend on the public health system, which is made up of government hospitals, rural health units, and local barangay health stations. About 40% of all hospitals in the Philippines are public and contribute to over half of all beds in the country [9]. Provincial governments maintain large provincial and district hospitals while municipal governments are in charge of rural health units and barangay health stations, which are primary health-care facilities. PhilHealth covers costs of qualified public and private health services for indigent members through PhilHealth's Sponsored Program. In 2006 there were close to 5 million indigent families, or 25 million individuals, enrolled in this program.

### The Visayas and health status of children in the region

While the National Health Sector Agenda was intended to be implemented nationwide, the DOH designated the Visayas, one of the three major island groups, as the site for QIDS implementation. The Visayan islands cover about one-third of the geographical area of the entire country. A main advantage of the Visayas in this study is the geographic isolation of many island sites, limiting cross-over of people between intervention sites. As most of the Visayas is rural, many of its residents are poor and depend on farming and fishing as their source of income [10,11]. Children in such rural areas of the Philippines have a high burden of disease. According to the 2003 National Demographic and Health Survey conducted by the Philippines Department of Health, infant mortality is 24 deaths per 1,000 for urban areas and 36 deaths per 1,000 in rural areas [12]. The national mortality rate for children under age 5 is 30 per 1,000 for urban areas and 52 per 1,000 in rural areas. From 2000 to 2003, diarrheal disease and pneumonia accounted for 25% of all deaths among children under age 5 [13]. Approximately 12% of children in rural areas have symptoms of acute respiratory illness, but only 43% of them were taken to a health facility or provider for treatment. Among those with diarrhea, only 29% were taken to a health facility or provider. Data from the 6th National Nutrition Survey reveal that 30% of Filipino children under age 5 are stunted (less than 2 standard deviations (SD) below the mean height for age), 27% are underweight (less than 2 SD below the mean weight for age) and 5.3% are wasted (less than 2 SD below the mean weight for height) [14].

## Results

To implement a social policy experiment required that we first recognize the research opportunity in a complex environment surrounded with scientific, bureaucratic and economic pressures. However, this, we found, was just the beginning. Establishing the scientific basis for evaluating policy, overcoming the unique challenges of randomization, carrying out the policy interventions and collecting community level data are all key elements that, in our experience, distinguished both the challenge of conducting a social experiment and the integrity of a policy experiment from clinical trials and other evaluation efforts. Herein we describe the vital factors that led to the successful implementation of the QIDS social policy experiment.

### Success in establishing QIDS – recognizing opportunity

At the outset, we realized two important facts about the broad reform agenda in the Philippines. The first was, despite global interest in health sector reform, there was little scientific information on the effectiveness of even the most basic reform policies considered in the Philippines and other parts of the world. In this case, there was a dearth of compelling evidence to show that increasing insurance coverage leads to improved health status in populations and an international debate over the benefits of pay-for-performance. Second, the Philippines government lacked the financial resources for a national roll-out of either proposed reform policies. The scientific uncertainty of the effectiveness of either policy, coupled with the practical problem of financing a large-scale introduction of the policies, was the opportunity that we recognized for putting a policy experiment in place.

Though conceived and spearheaded by Dr. Peabody of the Institute for Global Health at University of California San Francisco (UCSF) and Dr. Solon of the UPecon Foundation based at the University of the Philippines School of Economics, QIDS was designed in close relationship with the DOH and PhilHealth. We discussed at length, for example, the opportunity for a scientific study beginning with the then Secretary of Health, Dr. Alberto Romualdez. The DOH, recognizing the unique opportunities around informing policy and establishing global solidarity, brought PhilHealth into the discussion from the start, giving them a central role since PhilHealth is the payer for the national health insurance program and the institution that could best implement these reform policies. Through these discussions, a new type of collaborative was formed. We then linked the two broad HSRA reform goals–increasing access and improving quality–with the detailed, specific hypothesis-driven interventions and their experimental introduction.

### Funding and timing of the study

Funding is a ubiquitous issue particularly in health policy research where the costs are high and the need for action urgent. We had two main sources of funds: First, PhilHealth committed and maintained a financial obligation to the QIDS experiment by funding the actual policy interventions. This funding, although relatively modest, was critical to the overall credibility of the study. For the scientific efforts–ones that fall outside of most implementing agencies purview, we had to turn to outside sources. In the Philippines, or most other developing countries, there is no tradition of funding scientific research, thus external funding from international sources was pursued. We sought research money from the U.S. National Institutes for Health (NIH). Securing funds for the full 5-year study, however, proved to be a challenge, particularly given the highly competitive environment at the NIH. Concerns raised included the uncertainty in successfully carrying out such a large experiment requiring coordination with the government and the value of the study's findings outside of the Philippines. In due course, we successfully obtained an R01 grant from the National Institute of Child Health and Human Development (NICHD) for the 5-year study (R01-HDO42117), making the argument that the effects of pay-for-performance, which was undergoing a similar renaissance in the U.S., and universal coverage, being pursued through initiatives such as the State Children's Health Insurance Program, were poorly understood.

A related and formidable challenge was to time the introduction of the official policies with the arrival of the research funding. Our rigorous design *required *that we conduct a pre- and post-intervention assessment around the outcomes of interest. We timed the grant application to follow policy approval but to precede policy implementation. In part this was due to good planning and coordination with our partners at DOH and PhilHealth. Thus, while announcements were made early on, implementation was more flexible and could, in this case, be accelerated to meet our research funding schedule.

### Formalizing the experiment into the legal and regulatory systems

The policy environment from a methodical, scientific perspective is uncertain and fraught with unplanned events. In other policy reform settings, we had observed an evanescent enthusiasm accompanying research activities that, in the day-to-day context of institutional operations, would dissipate. Recognizing these realities we sought to minimize their impact. We thus obtained legislative and regulatory sign-off that codified the integration of the experimental design directly into the existing infrastructure. Specifically, QIDS did this early on by creating several formal partnerships–provincial, local and national–for the experiment. For the provincial partnerships, we executed legally binding Memorandums of Agreement (MOAs). To obtain buy-in at the local level, we extended these commitments down to the 30 participating local government units in the participating provinces, through additional MOAs obtained prior to site randomization. Once these agreements were signed, we vetted QIDS a second time with the national government (PhilHealth) and requested formal resolutions from the PhilHealth Executive Board.

To further minimize external events, PhilHealth played an operational role, both centrally and regionally, to implement the study. Through the partnership, the central office designated formal budget administrative responsibilities and the regional staff explicitly collaborated at the ground-level to implement the policy interventions, administer the clinical vignettes (which assessed the quality of care), disbursed the bonus payments, conducted some of the intervening data collection, and monitored insurance coverage levels.

### Randomizing the policies and controlling for experimental biases

In the Philippines, there are 220 districts, with an average population size of 250,000. Since the interventions were to be implemented at the provincial district level, an administrative unit that manages the delivery of health and other social services, we needed to randomize the experimental design to this level of the community. The two policy interventions, including controls, were thus randomly assigned to the 30 districts situated in the 11 provinces in the Visayas.

Randomization, proved to be complex, because there was an a priori assumption made by communities that participation in the study meant receiving a new policy with new resources. Accordingly, after the sites were identified, the study team held a series of meetings with government officials, provincial governors, provincial and municipal health officers, regional PhilHealth officers and the mayors (who were the heads of municipalities or cities), to seek their voluntarily consent to participate in the randomization and to provide them with the operational details pertaining to the implementation of policies and data collection.

We grouped districts with similar population characteristics into matched blocks of three before randomizing. The group characteristics, obtained from national census and local data, included population size, average income, labor force participation rate, functional literacy, infant mortality rate, maternal mortality rate, percentage of the population with insurance, proportion of the population that is rural, and proximity to Manila. To minimize within-block variation for each characteristic, the matched locations had to be within 10 to 25% of the existing variation. Randomization was then done among the matched blocks, with one site randomly chosen as an 'A' site, one as a 'B' site, and the third as a control or 'C' site, which are described in more detail below.

Because of the possibility that the study itself may alter the behavior response of the participants over time, careful consideration was given to control for potential participation effects in the study design. Because the study called for an evaluation of physician practice and children's health status for two clinical conditions, one effect might be that doctors change their practice patterns for the diseases in question. To overcome this, we selected or "screened-in" children who had been admitted with diarrhea or pneumonia. Screened-in children were identified as the subset of patients to be followed longitudinally. We also sampled children who were admitted for conditions other than diarrhea and pneumonia, or "screened-out". If there was a participation effect of the doctors, we would be able to measure it by comparing relative changes over time. Another potential participation bias was due to patient selection, wherein there might be substantive differences between children who came to the hospital versus the general population. We thus included a population-based survey of random households from the catchment areas surrounding QIDS hospitals.

### QIDS interventions

When we developed the policy interventions for the study, naturally, we wanted them to be linked closely to the areas that corresponded to the primary HSRA reforms. Our explicit task was to tie well-defined hypothesis-driven interventions to the actual HSRA policies aimed at improving health care access to the poor and increasing the quality of care. QIDS thus introduced expanded PhilHealth membership to children under 6 years of age and then measured changes in utilization of all services. We call this the Access Intervention carried out in the 'A' sites. PhilHealth membership in the A sites was made available at no cost to all indigent children under the age of 6 years and their families. In addition, this scheme typically results in 100% financial coverage for ordinary cases such as pneumonia and diarrhea; if charges exceed the PHIC benefit limits, they are shouldered by the local government unit hospital.

The second policy, a supply-side intervention, targeted physicians by introducing financial incentives for doctors providing high quality care, which is in essence a pay-for-performance scheme. We call this the Bonus Intervention, carried out in 'B' sites. Physicians in the district hospitals randomized to the B sites had to meet quality standards, which were pre-determined, to be eligible for bonus payments. We used a measurement standard of quality, referred to as Q*, wherein we combined an average of clinical vignette scores, which measured the quality of clinical care of doctors caring for patients with diarrhea and pneumonia, with a measure of facility case load, and a measure of patient satisfaction [15]. Each quarter, the QIDS Q* metric is computed from randomly selected physicians for each hospital assigned to this intervention and official PhilHealth issuances are made to hospitals indicating whether or not it is qualified to receive bonus payments. The bonus amount is calculated quarterly for qualifying hospitals and distributed to all clinical staff, with half going to the physicians, and the remaining half divided between nurses, administrators and support staff.

### Longitudinal and multi-level assessment of health determinants and outcomes

Data collection presented two distinct challenges relative to evidence generation: timeliness and comprehensiveness. We conducted a baseline, or pre-intervention, evaluation of study facilities and participants. Smaller data collection efforts were carried out at various intervals after the introduction of the policy interventions followed by a second major round of data collection after approximately two years. With these pre- and post-intervention assessments, we are able to observe transition states over time and other changes in variables of interest not possible from a cross-sectional study; this design also allows the control for otherwise-omitted variation in outcomes among individuals [16]. Our design and related data collection allow us to measure the impact of the policy interventions on outcomes of interest using difference-in-difference estimators, which we use to compare the changes (before and after intervention) in the outcomes in *intervention *sites with the corresponding changes in *control *sites. By looking at the changes over time we control for individual and area-specific characteristics and secular trends that might confound the estimated policy impact.

**Figure 1:**
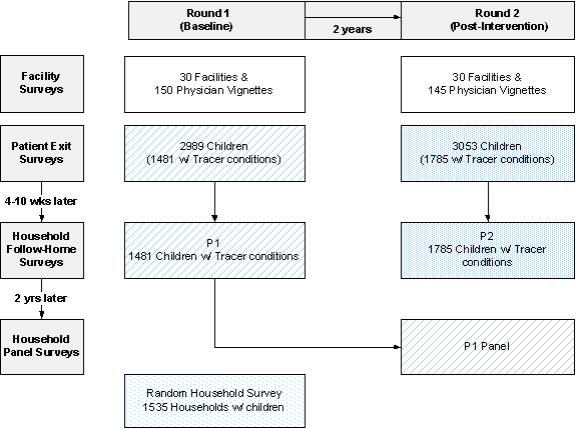
Multi-level data collection in the Quality Improvement Demonstration Study.

The second challenge to data collection was ensuring that we captured all changes, not just the outcomes of interest that might occur as a result of the policy interventions. Given the potential of the policy interventions to change provider and patient behaviors and outcomes, facilities and physicians, patients and their households were all included in the assessments. Figure 1 illustrates the multi-level nature of the study as well as its longitudinal design, which we briefly describe below.

The comprehensive data collection started with QIDS hospitals and physicians, surveyed on a semestral basis and on a quarterly basis respectively, giving numerous data points for longitudinal study. At each facility, structural measures, such as the availability of electricity and sanitation, overall cleanliness, qualified staff, organization of services, drugs, and equipment, were collected. However, while structural measures are necessary to monitor certain aspects of quality, they are insufficient indicators of care provision. Thus to assess the quality of care and accurately measure the patient-provider interaction, we used clinical vignettes. Vignettes, written case scenarios which simulate patient visits and are followed by questions for doctors to answer, are used as an accurate, affordable, and valid measure of a doctor's ability to evaluate, diagnose, and treat specific conditions [17-19]. Table 1 outlines the measures used at each assessment.

**Table 1:** Summary of Measures Collected for QIDS Assessments of Patients, Households, Physicians and Facilities

			**Household Survey**	
			
Measure	**Facility Survey**	**Patient Exit Survey**	**Follow-home**	**Panel**
**Quality of Care**				
Clinical Vignettes	X			
Structural survey of facility	X			
**Patient Response**				
Utilization and cost		X	X	X
Service characteristics		X	X	X
**Demographics**				
SES		X	X	X
Expenditures and charges		X	X	X
Home Observation and Measurement of the Environment (HOME)			X	X
**Subjective Health**				
General Self Reported Health		X	X	X
**Objective Health**				
Anthropometrics		X	X	X
Blood screening:				
Lead		X		X
Hemoglobin		X	X	X
Folate		X		X
C-reactive protein		X	X	X
**Cognitive Development**				
Bayley Scales of Infant Development			X	X
Wechsler Preschool and Primary Scales of Intelligence			X	X

Children at the QIDS sites were enrolled into the study upon hospital discharge, and extensive health assessments, including blood tests and subjective health assessments, were made at this visit, which we called the Patient Exit assessment. Our two tracer conditions, diarrhea and pneumonia, identified the subset of patients to be followed longitudinally. By examining a specific health problem or process, tracer conditions allowed us to focus on the impact of the policies on these particular conditions. Children screened-in with the tracer conditions were followed home 4 to 10 weeks post hospital discharge. Apart from detailed information on the household, blood, subjective health, and cognitive data were also collected during this home visit, which we called the Follow-Home Household assessment. These same patients were then followed two years after the initial hospital visit for another assessment of health and cognitive status in the Panel Household survey.

### Data collection team and data quality

Because of its breadth, QIDS required a large, well-qualified multidisciplinary data collection team. Accordingly, in each of the 11 study provinces QIDS assigned a team of three medical technologists, two psychologists and one supervisor. Supervisors are responsible for the timeliness and rigor of data collection and ensure that all eligible children are asked to participate, the survey visits are scheduled on time, facilitate team logistics and timely delivery of the biomarkers. Three regional program managers oversee the supervisors in the 11 provinces addressing concerns and issues raised by the field staff. Finally, central staff, consisting of a survey leader, a program manager, research assistants, and a data entry team, contribute to a data collection process that is efficient, on-time, and available.

As a result, QIDS has a high response rates for both initial and continued participation. In both rounds of data collection only about 10% of eligible participants refused to take part in the study. Of those who agreed to participate in the study, 0.5% refused at the time of the follow-home household survey. The refusal rate for panel children who were initially surveyed two years earlier was 1.5%. And between the two rounds of data collection less than 1% of children were lost to follow-up.

### Inter-current use of the QIDS data

A common, and valid, criticism of policy research is that results are not timely and accordingly often not relevant to policy-makers where the study is being conducted [20]. To overcome this concern, we conceived of using QIDS data to guide government initiatives in real-time. In 2006, for example, the original 1997 HSRA implementation strategy was revised under a new administration and called FOURmula One (F1) for Health. We did some analyses from the first round of QIDS that led to shifting the focus of F1 towards increasing PhilHealth enrollment, quality of care, and no-balance insurance billing. More recently, in 2007, the QIDS methodology and data were again critical inputs for the DOH in designing the Monitoring and Evaluation of the F1 agenda. QIDS analyses have also formed the basis for bilateral assistance from USAID, whose goal is to strengthen and institutionalize policy evaluation and coordinated policy reform. The QIDS framework has been used by the Philippine Government to demand impact evaluation for a host of other bilateral and multilateral projects the country. QIDS also produces policy briefs, published three to four times a year, to update the policy community on policy-relevant analysis. QIDS data are publicly available. All non-identifiable data along with questionnaires and data codebooks are in the public domain and available on the Internet [21]. The QIDS website also serves as a repository of findings from completed analyses.

## Discussion

This paper reports on the successful implementation of QIDS, a large policy experiment in the Philippines. We argue that policy should be evidence-based and thus there ought to be a strong interest in making the policy environment more amenable to scientific analyses. QIDS demonstrates that with a scientific framework, collaboration and coordination, opportunistic insights and some serendipity, large-scale policy experiments can be conducted. Below, we first discuss the importance of bringing evidence into policy making and its major challenges. Then we show how the lessons learned from QIDS might advance our collective experience with policy experiments and integrated science-based policy making. We conclude with a discussion on our limitations.

### Integrating evidence into policy making

Experience suggests that policy-making is typically devoid of scientific evidence and frequently not even supported by anecdotal evidence [22]. The evidence-base for policies, both inside and outside the health sector, is certainly weak when compared to evidence required for decision making in other areas, such as clinical medicine. This is often attributed to the complex nature of the policy environment [23,24]. First among these complexities is the breadth of social policies that, by nature, affect individuals and communities as a whole through a variety of disparate pathways [25]. Modeling or quantifying the impacts of these broad policies is necessarily a challenge. For instance, even more focused health policies are rarely evaluated because of the difficulties in monitoring and evaluating multiple activities, the challenges of observing disparate effects, or because there has been too little time to observe meaningful changes. Secondly, the "politics" of policy-making make for an environment where decision-makers are less interested in science, health impact or effectiveness, and more concerned with electoral interests, financial implications, and views of particular communities or groups. A related barrier to evidence-based policy-making are structural barriers to communication between researchers and decision makers, which include "differences in priorities, language, means of communication, integration of findings and definition of the final product of research" [4].

Despite the challenges to evidence-based policy making, the fact remains that governments need to know at the outset of policy development, and after policies have been implemented, the likely and achieved impacts in terms of both the positive and negative outcomes. The most rigorous way of monitoring the impact of an intervention or program, when feasible, is using a randomized controlled design, which assesses the impact of a policy by exposing a group of people to the policy intervention of interest at random while withholding the policy to a control group [16]. Baseline data are collected prior to the introduction of the policy, and again at an appropriately determined follow-up time. The differences between the measurements at baseline and follow-up estimate the net effect of the policy of interest, assuming proper randomization and large enough sample size to identify a minimum detectable effect [16]. Randomizing policies to whole communities, securing the units of analysis, maintaining internal consistency, timing the interventions with data collection, ensuring high quality data, and the inherently political nature of the evaluation process are all unique to the social experimental setting.

A notable example of a randomized policy study that arose from a governmental sponsored social investment in evidence-based policy is PROGRESA or Oportunidades in Mexico. Like QIDS, PROGRESA, was a major government initiative; aimed at reducing long-existing poverty and developing human capital within poor households [26]. Introduced in 1997, this was the first national controlled randomized anti-poverty intervention program in a developing country [27]. The intervention offered conditional cash transfers in order to promote incentives for positive behavior, such as participation in health and nutrition programs, including prenatal care, immunization, and nutrition supplementation, and provided incentives to promote children's school attendance. Communities were also randomly phased into treatment, allowing researchers to identify the impact of transfers on a variety of child health and educational outcomes. A key feature of the program was the opportunistic series of rigorous impact evaluations. While PROGRESA evaluations are considered some of the most expensive ever introduced by the government, they have been internationally acknowledged as being an invaluable source of data for governments in guiding policy decisions [28]. PROGRESA has subsequently expanded into the ongoing Oportunidades Program, which continues the evaluation practice. Similar to the experiences with PROGRESA, QIDS underscores the feasibility and value of studying policy interventions in a scientifically rigorous fashion and then using these data for large sector governmental changes in a systematic and integrated fashion.

### Lessons learned

Some of the lessons learned from QIDS might guide future research, help avoid pitfalls and foster interest in evidence-based policy research. We list here seven of the most important lessons we learned that formed the foundation of our work: 1) recognizing opportunity and forming a multi-institutional collaboration, 2) identifying and securing funding and timing the study with policy changes, 3) formalizing the study, 4) randomizing the policies and controlling for potential biases, 5) introducing sustainable policy interventions, 6) collecting comprehensive data, and 7) using results to inform policy-making in real-time. Those that are more complex are described in more detail below.

First, we found opportunity in the Philippines health reform process in the scientific uncertainty surrounding the effectiveness of the policies in question. Since few policies have been scientifically validated, there are likely many opportunities to be found to assess health impacts [25]. Although there is always serendipity around opportunity, policy analysis lends itself to opportunistic evaluations if for no other reason than this dearth of scientific data.

The insight, we feel, is to be able to recognize opportunity and then be prepared to act on it in timely manner, which requires experience in the policy environment, relationships with key stakeholders, and familiarity with the scientific literature. Large-scale policy studies by construct require scientific expertise and objectivity that is provided by non-political entities such as academic institutions. However, academicians typically separate themselves from the political arena and often find themselves working in a setting devoid of real-world context or experience [20]. We argue that those involved in research ought to cultivate familiarity with the policy environment and build relationships with policy-makers and stakeholders. Likewise, being armed with scientific background is a key element in being prepared to recognize opportunities as well as for planning and ultimately implementing policy studies. As our experience shows, opportunity may also flow from financial impediments that prohibit large-scale implementation of policies of interest.

Not surprisingly, we found that success requires a high degree of multi-institutional collaboration, involving government entities and the private entrepreneurship of the investigators. Our partnership involved disparate institutions: the Philippines Department of Health, which has a strong relationship as well as some rivalries with PhilHealth, the National Insurance payer, and our two academic institutions. Our collaboration was not always easy; however it was rooted in dedicated leadership committed to improving health and advancing research. Leadership, we observed, also involved a significant level of risk taking from both the policy makers and researchers involved. For policy makers, there is risk in that the partnership or experiment might fail to materialize, be funded, or be implemented. In QIDS, the Philippine government had to trust their academic counterparts, who ultimately could not control the outcomes, and thus risked their own political capital by sponsoring a study that might uncover effects of government policy that were not flattering or even become the source of eventual embarrassment. From the perspective of the academic leadership, the risks stem from the limited control of the policy environment and the inability to create a stable setting that is the usual basis for a scientific study. There is additional professional risk associated with the opportunity costs of pursuing an opportunity that may never materialize, thus hurting chances for promotion or advancement. Arguably, research funding for policy evaluation is harder to obtain, the costs typically are high and there are legitimate concerns over the generizability of the ultimate findings. Accordingly, given the risks involved, the early dialog between the collaborating entities necessitates understanding and trust between partners.

In our experience, tensions may arise when collaborating entities that have varying levels of financial investment in the study. Perhaps conflicts can be discouraged if costs are shouldered equally or if a neutral outside source provides the necessary financial resources, but this seems unlikely to happen in the uncertain funding environments that characterize competitive research. In the developing country setting in particular, co-funding is almost always necessary to conduct these types of large-scale policy experiments. To ensure scientific integrity, study funding should come from donor agencies free of conflicts of interest with participating entities. Securing these funds, for example from the U.S. National Institutes of Health (NIH), bestows legitimacy as well. The challenge is to succeed in the highly competitive environment, and a good degree of luck is involved in this process. Ultimately, success will be largely dependent on the ability to delineate or distinguish between plan and implementation. Researchers have thus proposed an International NIH to overcome the challenges of finding funds for research in less developed countries [29]. Lastly, as we described in our Results, the funding stream must be timed with the introduction of the interventions as well as data collection. Probably the only assurance would be to secure funding ahead of formal approvals for the study, which requires a great deal of work and perhaps serendipity on the part of the investigators to coordinate.

Though partnerships and funding are the building blocks of the policy study, a key finding from QIDS that might be overlooked is the formalization and legalization of the policy interventions into the existing health care system. We find that this integration is required to maintain security in what may be a highly uncertain or changing bureaucratic environment. It is not untypical in developing countries that research endeavors fail due to shifts in political parties, changes in agency heads, or economic downturn [30]. We found that formal board resolutions and signed legal documents protected us against unforeseen events.

With agreements in place, randomizing communities is another achievable step in the policy experiment; so is introducing a control arm. Voluntary participation is not only a cornerstone of research, but essential in a policy environment that will likely become even more complex once the study is underway. Randomizing populations to interventions is always contentious, particularly in an intervention-control design where some communities will 'not get something'. We anticipated and certainly experienced reluctance at the district level to randomization to the control arm, mainly by hospital directors. We ultimately overcame this hurdle by describing the control arm to participants as part of ongoing change justifiable given the introduction of other policies in an environment that was not strategically or operationally static in the long term. Our discussions at the local, provincial and national levels explaining the design ex ante were key in QIDS.

Another important lesson is to carefully choose interventions so that they are relevant, implementable, and can eventually be scaled-up for large-scale introduction. Because the HSRA goals were rather broad, we decided to introduce specific policies that could be later be easily introduced at the national level by the DOH and PhilHealth. Deciding the details of these interventions required us to have a good deal of knowledge regarding PhilHealth's capabilities and capacity for policy change. Through communications with PhilHealth, it was decided that we would focus on children under six years of age, where we were likely to see the greatest impact in the shortest time frame and in a population that would receive the greatest amount of political support. However, a criterion set by PhilHealth was that a child must be a dependent of a PhilHealth member in order to take advantage of expanded insurance benefits. Thus, we felt it was necessary, as part of this intervention for QIDS to take additional measures to enroll entire families into PhilHealth and set goals for enrolling a certain number of families. These goals were individualized with each mayor and then formal arrangements were put in place to achieve them. This strategy demonstrated to be cost-effective at $0.86 USD per enrollee, making it an inexpensive yet effective approach that can be scaled-up by PhilHealth [31].

Similarly, we designed our Bonus Intervention in a way that could also be easily expanded by PhilHealth. This intervention introduced a performance-based payment scheme for hospitals and physicians based on a quality of care performance measure which we refer to as Q*, a metric that measures clinical performance, patient satisfaction and volume of physician services. We found that the Q* measure was readily introduced into our 30 different study sites distributed over a wide-geographic and disparate-cultural area, largely because data collection did not require a great deal of marginal resources [15]. Data on Q* component indicators are collected through three straightforward mechanisms. The clear transparency of the measurement and issuances based on Q* through the notification of provincial governors, hospital directors and physicians proved to enhance credibility and support for the data collection. This measure is now used for internal monitoring and to evaluate new approaches for improving performance. As Q* measurements are tied to bonus payments to the hospitals, it has also given the metric meaning and consequence. As with enrollment, the quality measurement has proven to be inexpensive and implementable, thus making it more likely to be expanded at the national level.

We believe that comprehensive data collection is critical to informing large-scale policy changes. Beyond the obvious, which is the need to collect data to evaluate the efficacy of the policies and the demonstration that multilevel data can be collected around a policy evaluation, there are other benefits of comprehensive data collection. First, there may be unexpected findings. In the QIDS study so far we have found a high prevalence of elevated blood lead levels among children that was previously unknown, unexpected prescribing practices among physicians, and a negative impact of extra budgetary insurance funding for the poor [31-33]. Second, with the public release of the data, there now exists a substantial learning opportunities around such a rich data set for other investigators in the Philippines and internationally. The data has attracted a number of investigators and continues to draw more.

Perhaps, most importantly, QIDS data has given rise to a deeper commitment to the health sector reform agenda. And it has informed policy-making in real-time. While health policy by its very nature is dynamic, research, by contrast, is slow to provide real time guidance to policy makers [34]. To surmount this, we partnered QIDS with governmental policy monitoring and evaluation (referred to as ME3), developed policy briefs and other activities to integrate research in a time-conscious manner. Monitoring and evaluation systematically monitors the progress towards targets and evaluation of policy outcomes within the policy cycle, which are key components of evidence-based policy making [28]. It has gained attention as a means to enhance sound governance by providing input into decision-making, including the budget process, providing information to support evidence-based policy discussions, and for understanding performance, critical for learning, planning and growth [35]. ME3, using QIDS methodology and data, will form the basis for real-time guidance to the Secretary of Health in assessments of how the health reform policies benefit the Filipino people.

### Limitations

There are a number of study limitations that warrant mention here. The first is that we are challenged by the study size in our hierarchically-designed study in three ways: While we were able to include a large sample of children in the study, we would have liked to include more communities and thus improved our overall sample size. With a larger number of units to randomize, we would be able to better assess policy impacts in differing settings and to have larger power in our statistical analyses. Similarly, while we were able to include a random household sample at our baseline assessment, we did not have the financial resources to conduct a second random household survey during the second round of data collection. Lastly, while we are able to follow our screened-in children and facilities as a whole over time and create a panel data set, our other data, such as physician vignettes, are obtained through serial cross-sections conducted every six months. This makes it difficult to follow specific providers over time and to link providers with children except at the facility level.

Lastly, although we have regularly communicated our results with key stakeholders and partners, for example PhilHealth, we are limited by their absorptive capacity to translate the results into timely action. This is perhaps best exemplified in our serendipitous finding of a lead epidemic among children in the Visayas–although our findings have been well communicated with department heads at the Department of Health, PhilHealth, and local health organizations, little action has been taken to deal with this problem.

## Conclusion

One of the biggest challenges to evidence-based policy making is balancing scientific evidence with context. The policy environment is slanted away from evidence and toward context or the social milieu, driven largely by financial constraints [34]. Apart from this, policy-makers often rely heavily on personal experience, local information, expert opinions, and pressure from advocates [36]. Clearly, policy made devoid of context runs the risk of failure. However, it is possible that there is a balance between evidence and context [34]. One of the ways to achieve this balance is by bringing together science with policy-making and engaging researchers and decision-makers in the study process. QIDS shows that evidence-based policy making done in conjunction with the goals and objectives of the governmental reform agenda can marry science with context.

As we have described, through collaboration with government decision-makers a large, prospective, randomized controlled policy experiment was successfully implemented at the national level as part of sectoral reform. While we believe policy experiments such as this can be used to inform health policy, our experience highlights the importance of key factors in success: being opportunistic, building strong collaborative relationships, timing the policy interventions with data collection, providing real-time feedback to policymakers during the course of the study, and integrating the study into the existing delivery of care infrastructure.

QIDS nurtures the growing attitude that translation of scientific findings from the bedside to the community can be done successfully and raises the bar on project evaluation and the policy-making process.

## Competing interests

The author(s) declare that they have no competing interests.

## Authors' contributions

RS participated in the conceptualization and drafting of this manuscript. JP was involved in the conception of the study and in the conceptualization and drafting of this manuscript. SQ participated in the study and helped draft the manuscript. OS was involved in the conception of the study and the drafting parts of this manuscript. All authors have read and approved the final manuscript.
